# Utilizing a Terrestrial Laser Scanner for 3D Luminance Measurement of Indoor Environments

**DOI:** 10.3390/jimaging7050085

**Published:** 2021-05-10

**Authors:** Matti Kurkela, Mikko Maksimainen, Arttu Julin, Toni Rantanen, Juho-Pekka Virtanen, Juha Hyyppä, Matti Tapio Vaaja, Hannu Hyyppä

**Affiliations:** 1 Department of Built Environment, School of Engineering, Aalto University, FI-00076 Aalto, Finland; mikko.maksimainen@aalto.fi (M.M.); arttu.julin@aalto.fi (A.J.); toni.rantanen@aalto.fi (T.R.); juho-pekka.virtanen@aalto.fi (J.-P.V.); matti.t.vaaja@aalto.fi (M.T.V.); hannu.hyyppa@aalto.fi (H.H.); 2 Finnish Geospatial Research Institute FGI, Geodeetinrinne 2, FI-02430 Masala, Finland; juha.hyyppa@nls.fi

**Keywords:** luminance measurement, lighting distribution, 360°, HDR imaging, 3D, terrestrial laser scanning

## Abstract

We aim to present a method to measure 3D luminance point clouds by applying the integrated high dynamic range (HDR) panoramic camera system of a terrestrial laser scanning (TLS) instrument for performing luminance measurements simultaneously with laser scanning. We present the luminance calibration of a laser scanner and assess the accuracy, color measurement properties, and dynamic range of luminance measurement achieved in the laboratory environment. In addition, we demonstrate the 3D luminance measuring process through a case study with a luminance-calibrated laser scanner. The presented method can be utilized directly as the luminance data source. A terrestrial laser scanner can be prepared, characterized, and calibrated to apply it to the simultaneous measurement of both geometry and luminance. We discuss the state and limitations of contemporary TLS technology for luminance measuring.

## 1. Introduction

Laser scanning is a commonly applied 3D measuring technology for indoor measurement. Laser scanning is based on measuring 3D coordinates from an environment using a laser beam. As a result, a 3D point cloud is formed from a dense set of 3D measurements. Most contemporary laser scanners also contain one or more integrated cameras that are used to capture a panoramic image used for point colorization. The R (red), G (green), and B (blue) values of the captured image are projected onto the point cloud to obtain coloring for points. In addition to visualization, the color information has been applied for registration [1] and segmentation [2]. However, the point cloud colorization quality varies, depending on the selected terrestrial laser scanning (TLS) instrument [3].

In the past, terrestrial laser scanning has been widely applied in archaeology [4], cultural heritage [5], forestry [6], industry [7], geology [8], surveying [9], and construction engineering [10]. Today, terrestrial laser scanners are also a commonly used instrument in the architecture, engineering, construction, owner, operator (AECOO) industry. In TLS, one path of development is automating the processing of raw measurement into more sophisticated 3D models [11,12,13]. Another path of development is the integration of parallel data and sensors in laser scanning [14,15].

Two-dimensional luminance photometry is commonly applied to measure indoor surface luminances [16,17]. Luminance is the measure of light reflected or emitted from an area, commonly measured in candelas per square meter (cd·m${}^{-2}$). In lighting design, luminance distribution is an important aspect, as it affects the security, well-being, visual comfort [18], and aesthetics of the indoor environment. The luminance distribution is usually measured via imaging luminance photometry, where a calibrated digital camera is used to obtain an absolute luminance value for each pixel. Imaging luminance photometry has been applied in the assessment of light pollution [19,20]. High dynamic range (HDR) imaging is a key technology in imaging luminance photometry [21]. In HDR imaging, a set of images with different exposure times is combined to extend the dynamic range of a single exposure. This technique has been applied in architecture [22]. Moreover, the HDR technique is under constant development, for example by being applied to 360${}^{\circ }$ imaging [23] and by improved image fusion algorithms [24]. As a technology, imaging luminance photometry via HDR imaging has become well-established. However, an innate problem in measurement relying on individual images is the loss of 3D data in measuring.

Via photogrammetric 3D reconstruction, 2D luminance images can also be utilized for obtaining a 3D luminance measuring of a measured indoor environment [25]. Still, photogrammetry can perform poorly when measuring the 3D geometry of smooth, mono-colored, and uniform surfaces [26]. Luminance measurement applications require accurate radiometric data, and the use of 3D luminance measuring in design would be beneficial not only for lighting designers but also for architects [27]. However, indoor 3D luminance measurements made with a terrestrial laser scanner have not been extensively studied. Existing research has shown that luminance maps obtained via imaging luminance photometry can be combined with TLS [28] and MLS point clouds [29,30]. As stated, contemporary TLS instruments commonly contain imaging sensors for point cloud colorization. As the sensors are increasingly applicable for HDR imaging [3], the utilization of such HDR imaging-capable TLS instruments for producing a 3D point cloud with luminance information is a topical development issue. While the use of TLS for lighting design via luminance measuring has been suggested in earlier research [27,28], a solution employing the TLS images for luminance measuring is missing, since Rodrique et al. [27] utilized a separate imaging luminance photometer and they did not register the luminance values into a 3D luminance point cloud. Instead, they assessed the geometry and luminance measuring as separate entities. Vaaja et al. [28] manually combined images obtained with a conventional single-lens reflex camera into a point cloud produced by TLS. However, in this case, the images did not cover the full 360${}^{\circ }$, and the data integration relied on manual methodology, limiting the efficiency.

In this study, we aim to present a method to measure 3D luminance point clouds. We apply the integrated high dynamic range (HDR) panoramic camera system of a TLS instrument for 3D HDR luminance measurements simultaneously with laser scanning. We present a method for utilizing the images captured with a TLS instrument as the luminance data source (Table 1). Firstly, we present the luminance calibration of a laser scanner, and we assess the accuracy, color measurement properties, and dynamic range of luminance measurement achieved in a laboratory environment. Secondly, we demonstrate the 3D luminance measuring process through a case study with a luminance-calibrated laser scanner. We analyze the results and discuss the effect of scanning angles on luminance measurements. In addition, we explore future research directions in 3D luminance measuring. The novelty of our study is that the method covers the 360${}^{\circ }$ 3D luminance measurements and increases the level of automation in the data integration. In addition, the luminance point cloud data is enriched with the angle between the surface normal and the measurement direction.

## 2. Materials and Methods

### 2.1. Terrestrial Laser Scanner

**Figure 1:**
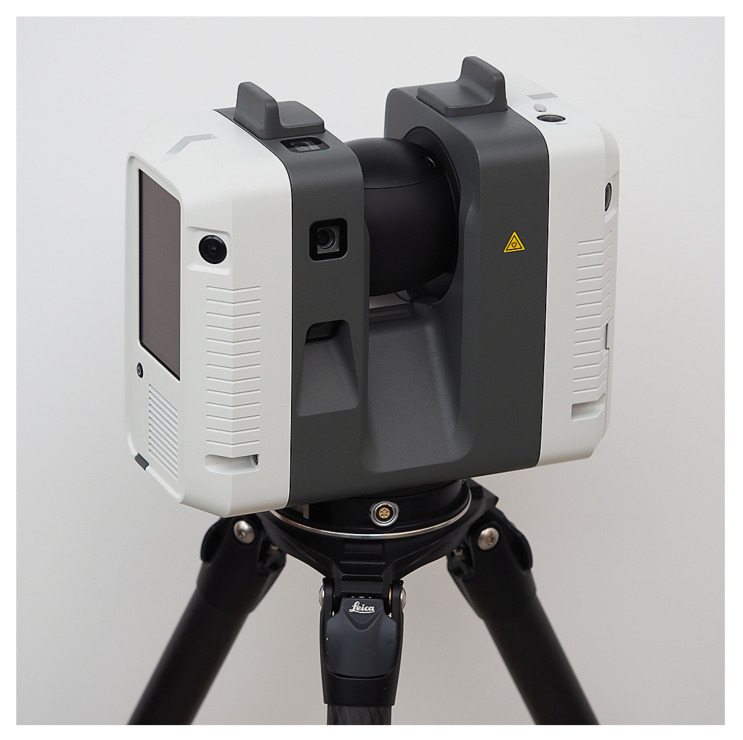
Time-of-flight scanner Leica RTC360.

For terrestrial laser scanning, we used a time-of-flight scanner Leica RTC360 (Hexagon AB, Stockholm, Sweden) [31,32]. According to the manufacturer, the scanning field of view is 360${}^{\circ }$ horizontal and 300${}^{\circ }$ vertical, and the measured 3D point accuracy is 1.9 mm at 10 m. The scanner has three 4000 × 3000 pixel image sensors mounted to the scanner body (see Figure 1). Together, the sensors cover a vertical view of 300${}^{\circ }$. These sensors are used to create a panoramic image of 20,480 × 10,240 pixels with 5-bracket HDR imaging. The entire equirectangular panoramic image consists of 12 adjacent vertical images. The total scan time is 4 min 21 s, including HDR imaging with a scan resolution setting of 3.0 mm at 10 m.

In the RTC360, HDR imaging is performed with a fixed exposure without any prior exposure measurements [3]. The imaging system of the RTC360 can therefore be calibrated to interpret the absolute luminance values of the measured environment. Furthermore, the panoramic image can be exported for editing as an EXR file without losing the high dynamic range of the images and registered into the point cloud without losing the dynamic information. These attributes make the Leica RTC360 a usable measurement device for luminance-calibrated terrestrial laser scanning.

### 2.2. Luminance Calibration of a Terrestrial Laser Scanner

#### 2.2.1. Reference Color Target

**Figure 2:**
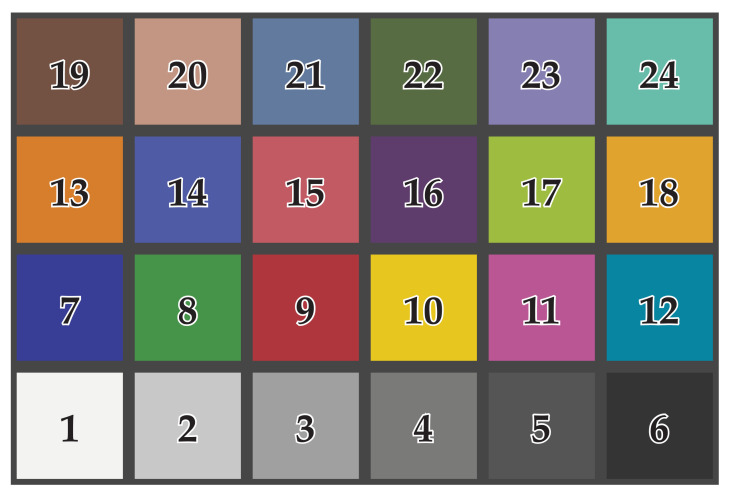
The measured target X-Rite ColorChecker Classic and the patch numbers used in this study.

A standardized color target, the X-Rite ColorChecker Classic chart (Grand Rapids, MI, USA) [33], was attached to the wall. The ColorChecker Classic chart is used in photography for creating camera profiles and correcting white balance and color. The chart is designed for color management in a variety of lighting conditions. Figure 2 shows the chart of 24 different colored patches with measured colorimetric reference data provided by X-Rite. The size of the ColorChecker Classic was 21.59 × 27.94 cm. In the X-Rite documents, the patches were labeled in a different order. Table 2 lists colorimetric reference data for the ColorChecker Classic manufactured after November 2014. The values were reported as CIE L*a*b* data.

#### 2.2.2. Reference Luminance Measurements for the Color Target

The 16-bit sRGB values were measured and calculated for each patch in the reference color target. This was done for two reasons. Firstly, 16-bit sRGB values were not provided by the color target manufacturer. Secondly, by measuring and calculating the sRGB values for each patch ourselves, we were able to obtain the exact measurements in our laboratory environment, including especially the influence of lighting. Reference luminance values from the X-Rite ColorChecker Classic were measured with a Konica Minolta CS-2000 spectroradiometer (Teban Gardens Cres, Singapore). According to the manufacturer, the range of measurable luminances of the spectroradiometer is 0.003–500,000 cd·m${}^{-2}$ with a luminance measurement accuracy of ±2%. For each measured patch of the color target, the average of five consecutive measurements was used. For each channel, every measured value was scaled to the maximum 16-bit sRGB, calculated from the CIELAB values provided by X-Rite [33].

**Figure 3:**
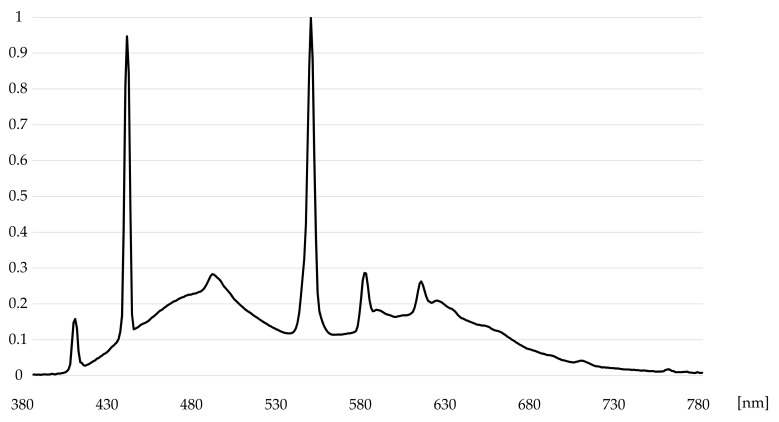
The normalized spectral power function of patch No. 1.

**Figure 4:**
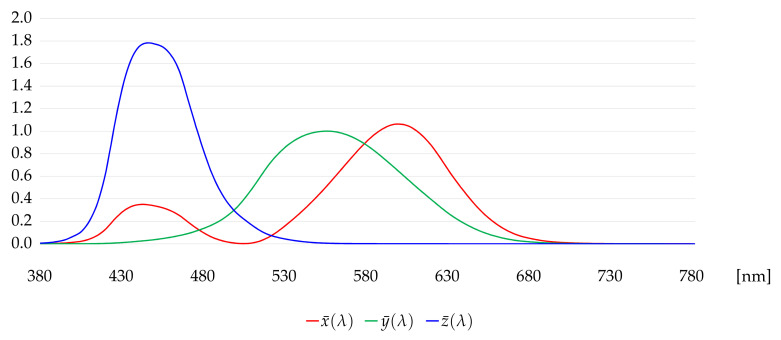
The CIE color matching functions $\bar{x}\left(\lambda \right)$, $\bar{y}\left(\lambda \right)$, $\bar{z}\left(\lambda \right)$.

A test environment was set up for measuring the radiometric capability of tripod-mounted TLS instruments (Aalto University, Espoo, Finland). The space was illuminated by luminaires fitted with D65 standard fluorescent tubes with a color rendering value R_a_ > 93. Figure 3 illustrates a spectrum of the patch number 1 (Figure 2) in the ColorChecker measured with the spectroradiometer. The spikes of the D65 fluorescent illuminant are clearly visible in the spectrum. Figure 4 illustrates the CIE color matching functions $\bar{x}\left(\lambda \right)$, $\bar{y}\left(\lambda \right)$, $\bar{z}\left(\lambda \right)$ [34].

Each spectral power distribution P(λ) of the measured patches was converted into *X*, *Y*, and *Z* colour values applying the CIE color-matching functions [34] $\bar{x}\left(\lambda \right)$, $\bar{y}\left(\lambda \right)$, $\bar{z}\left(\lambda \right)$ (Equations (1)–(3)):

(1) $$X=\int P\left(\lambda \right)\bar{x}\left(\lambda \right)d\lambda ,$$

(2) $$Y=\int P\left(\lambda \right)\bar{y}\left(\lambda \right)d\lambda ,$$

(3) $$Z=\int P\left(\lambda \right)\bar{z}\left(\lambda \right)d\lambda ,$$

For each patch, the *X*, *Y*, and *Z* values were normalized and then converted into linear *R*, *G*, and *B* values in the sRGB (IEC 1999) color space, applying Equation (4):

(4) $$\left[\begin{array}{c} R_{linear}\\ G_{linear}\\ B_{linear} \end{array}\right]=\left[\begin{array}{ccc} \;\;\;3.2406 & -1.5372 & -0.4986\\ -0.9689 & \;\;\;\;1.8758 & \;\;\;\;0.0415\\ \;\;\;\;0.0557 & -0.2040 & \;\;\;\;1.0570 \end{array}\right]\left[\begin{array}{c} X_{D65}\\ Y_{D65}\\ Z_{D65} \end{array}\right]$$

The acquired linear *R*, *G*, and *B* values were then scaled to make them comparable with the reference values and then applied in order to calculate the relative luminance values with Equation (5) from the sRGB standard [35]:

(5) $$L_{r}=0.2126R+0.7152G+0.0722B$$

#### 2.2.3. Characterizing the Color and Luminance Capturing of the TLS

**Figure 5:**
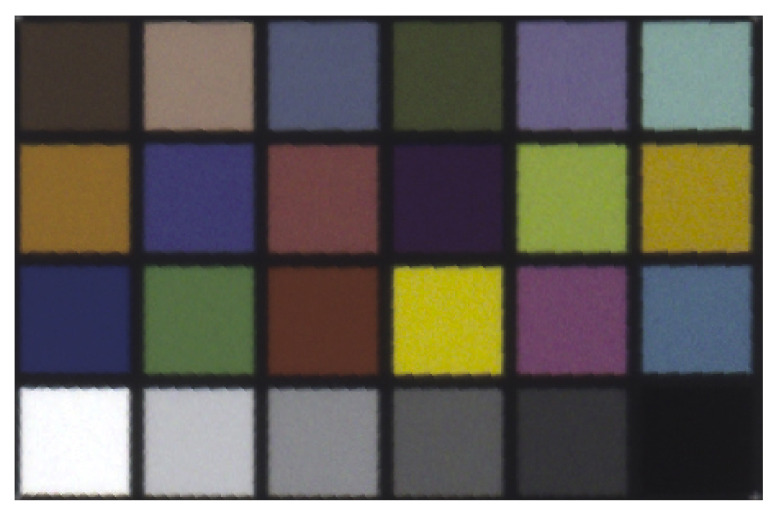
The color target cropped from the panoramic image captured with the TLS instrument.

The HDR images (Figure 5) captured with the TLS were first exported as 32-bit EXR files which were then converted to linear 16-bit TIF format. From the linear images, the sRGB (standard Red Green Blue) *R*, *G*, and *B* values were obtained as a median pixel value for each patch of the color target and as the average of five images. The values were scaled in order to make them comparable with the measured values. For each channel, every value measured with the TLS was scaled to the maximum 16-bit sRGB calculated from the CIE L*a*b* values provided by X-Rite (Table 2). The 16-bit *R*, *G*, and *B* values were then converted into relative luminances applying Equation (5). A luminance calibration factor was obtained by comparing the relative luminance measured with the TLS to the absolute luminance measured with the spectroradiometer.

### 2.3. Luminance Data Processing

**Figure 6:**
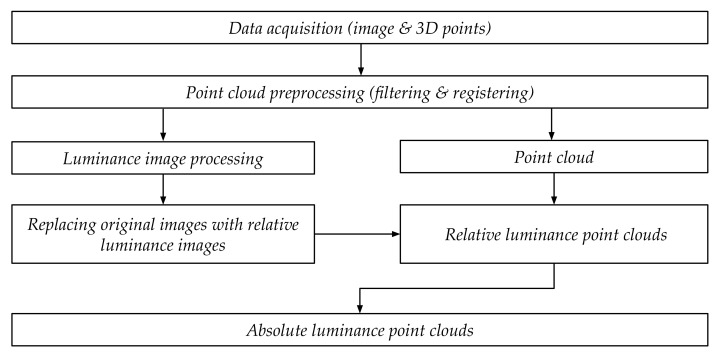
The workflow for creating data for indoor 3D luminance maps.

As in Section 2.2.3, the HDR images were exported as 32-bit EXR files from the TLS measurement data, and the 32-bit EXR files were converted to 16-bit TIF files, applying Python 3.6.9 with libraries OpenEXR (1.3.2) (San Francisco, CA, USA), Numpy (1.16.6) (Cambridge, MA, USA), and OpenCV-Python (4.2.0.32) (Willow Garage, Menlo Park, CA, USA). Relative luminance values were calculated for each pixel in the 16-bit TIF files applying Equation (5), and the 16-bit relative monochromatic luminance values were coded over the three 8-bit RGB channels of a respective pixel and a new image was saved as an 8-bit TIF [25]. Hence, the new 8-bit relative luminance TIF image contains a wider dynamic range than a regular 8-bit RGB image, as all three channels carry the relative luminance data. The coded 8-bit file format allowed further processing of data in software that does not support a wider dynamic range, e.g., 16-bit data. The 8-bit TIF images were projected and registered as the *R*, *G*, and *B* values in the point cloud. Point by point, the *R*, *G*, and *B* values were converted back to relative luminance values. Finally, the luminance calibration factor (see Section 2.2.3) was applied to interpret the relative luminance values as absolute luminance values, and the absolute luminance value was registered to each point in the point cloud. Figure 6 illustrates the luminance point cloud generating process.

### 2.4. Case Study

#### 2.4.1. Study Area

**Figure 7:**
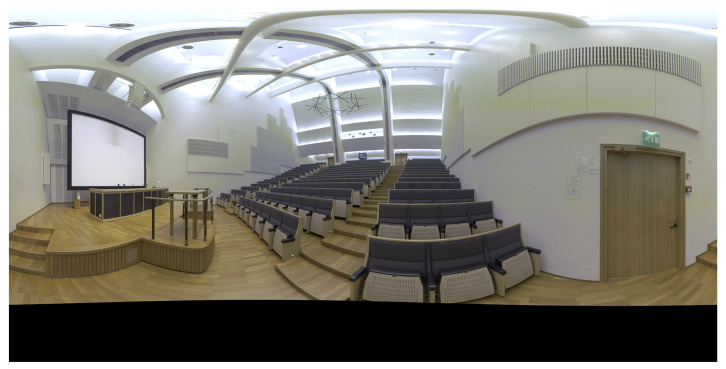
The 360${}^{\circ }$ panoramic image taken with the TLS instrument.

Figure 7 shows the space measured, the B-Hall, a lecture hall at Aalto University, Espoo, Finland. The maximum capacity of B-Hall is 320 persons, and the floor area is 297 m^2^. The lecture hall was illuminated only by interior lights.

Seven scans were taken from the hall, and the scanned point clouds were registered with the manufacturer’s Leica Cyclone REGISTER 360 version 1.6.2 (Hexagon AB, Stockholm, Sweden) software [36]. Each scan took 4 min and 21 s. Linear EXR images were exported as separate linear image files and converted to 16-bit TIF images. The scanned point clouds were colored with TIF images, and the color values of the point clouds were converted to absolute luminance values, as described in Section 2.3. Lighting analysis was performed with CloudCompare 2.10.2 software (EDF, Paris, France) with standard tools such as plane fitting, octree subsampling, and distribution fitting.

In laser scanning, the point densities of measured surfaces vary, depending on the different angles of incident and the distance from the laser scanner. Hence, in order to balance the point density, all the point clouds from individual scan stations were sampled in CloudCompare using octree-based subsampling, where the octree level was set to 12. The size of a single scan was about 160 million points, and subsampling reduced the point cloud to about 16–25% of the original. The densest point spacing of the subsampled cloud was about 5 mm. The subsampled point clouds were then merged into a single point cloud, and the merged point cloud was resubsampled with octree level 12 to avoid unnecessarily large file sizes.

#### 2.4.2. Sample Areas

**Figure 8:**
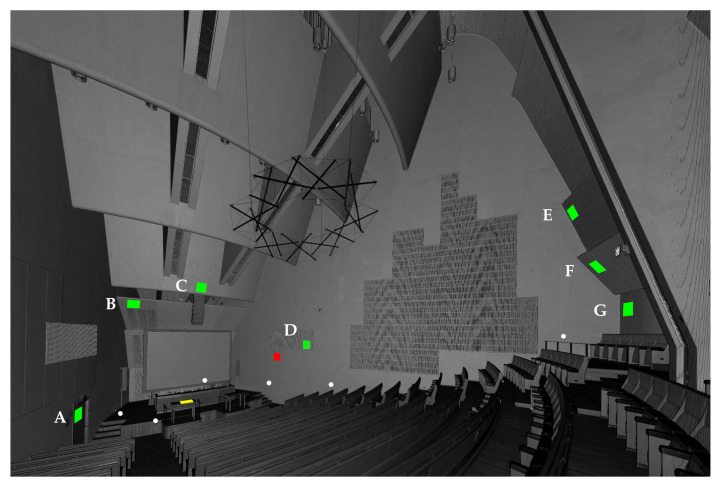
The intensity image from scanning station 6 shows the locations of the sample areas for luminance measurements. The green areas represent the sample areas A–G. The red area represents the vertical sample area. The yellow area represents the horizontal sample area. White points represent the 6 different scanning locations with the seventh scanning location being the observer of the image.

We chose two sample areas (horizontal and vertical) for detailed analysis. In addition, we present a concise analysis for seven sample areas A–G (Table 3). The sizes of the sample areas were 0.5 m × 0.5 m. Figure 8 presents the sample areas. We applied the CloudCompare 2.10.2 plane fitting tool in order to obtain the angles between the scan stations and surface normal. The angles between the scan stations and the surface normal of the sample areas ranged from 9 to 88 degrees. Detailed information on the vertical and the horizontal sample areas can be found in Appendix A.2.

## 3. Results

### 3.1. Reference Color Target Measurements

Table 4 presents the reference sRGB values measured from the X-Rite ColorChecker Classic with the spectroradiometer. The measured spectral power distributions were converted into sRGB values applying Equations (1)–(4).

### 3.2. Luminance Measurement Comparison

Table 5 presents the laser scanner luminance measurements compared to the spectroradiometer luminance measurements. Only the lowest row of grayscale patches (1–6) were used for luminance calibration (see Figure 1). The 16-bit values were calculated into relative luminance values, applying Equation (5). The laser scanner absolute luminance measurements were derived using a simple linear regression with the spectroradiometer values. We assume that the sensor noise increases the low-end luminance values captured by the camera of the laser scanner. Hence, an improved iteration of the laser scanner absolute luminance values was derived by reducing the original 16-bit value by the absolute difference in the smallest compared luminance value (18.2 cd·m${}^{-2}$− 13.9 cd·m${}^{-2}$ = 4.3 cd·m${}^{-2}$) multiplied by the calibration factor (146.3) obtained with the linear regression.

**Figure 9:**
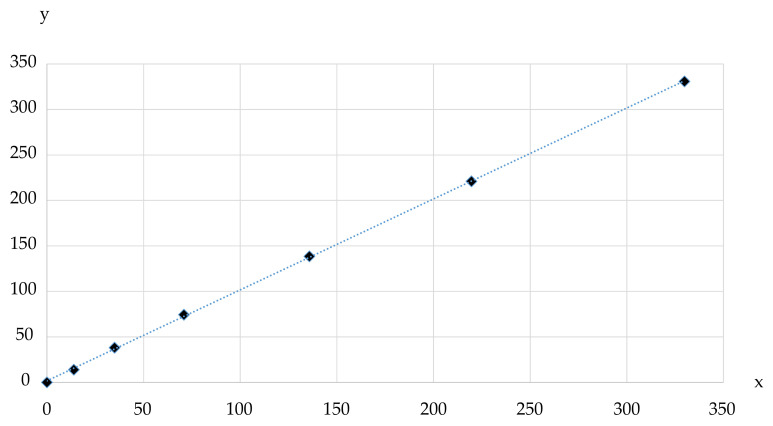
The TLS luminance measurement (y) presented as a function of the reference luminance measurement (x) and its linear trendline.

Figure 9 presents the laser scanner luminance measurement as a function of the reference luminance measurement and its linear trendline.

Applying the linear regression and the noise removal, the minimum and maximum measurable luminance values are 4.3 cd·m${}^{-2}$ and 443.6 cd·m${}^{-2}$, respectively.

Table 6 presents the consistency of five consecutive images captured with the TLS. The relative standard deviation (RSD) in the 5 repetitions was less than 2% for every grayscale patch of the reference color target.

The channel-wise values can be found in Appendix A.1. For the measured patch number 12, the processing from the spectrum into sRGB values resulted in a negative value for the red channel. This is an expected outcome for certain colors. However, it obviously makes the comparison between the measured values and the image values questionable to a certain extent. Furthermore, some measured color patches were very out of proportion in the image compared to the measured values. However, the large relative differences in single channels did not carry through to the calculated luminance values and their relative differences. This may be explained by the fact that often the large relative difference in a single channel was due to a comparison to values that were absolutely small.

Table 7 displays the relative luminance values calculated from the TLS 16-bit linear images compared to the reference values measured with a spectroradiometer.

**Figure 10:**
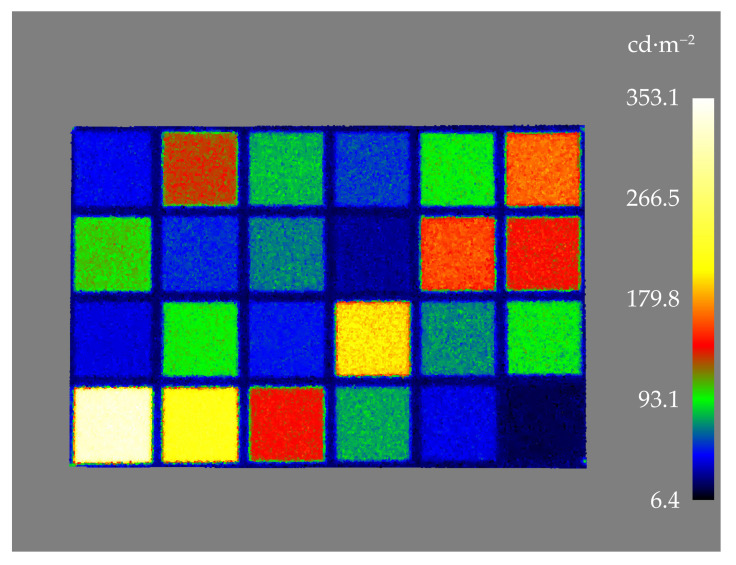
Luminances of the measured color target X-Rite ColorChecker Classic. The patches in the lowest row of patches (1–6) are the grayscale patches used for luminance calibration.

Table 8 presents the adjusted absolute luminance values for each patch in the color target measured with the TLS compared to the reference luminance values measured with the spectroradiometer. Figure 10 presents the 3D luminance point cloud of the reference color target.

### 3.3. Case Study

#### 3.3.1. The Case Study of a Luminance Measurements

**Figure 11:**
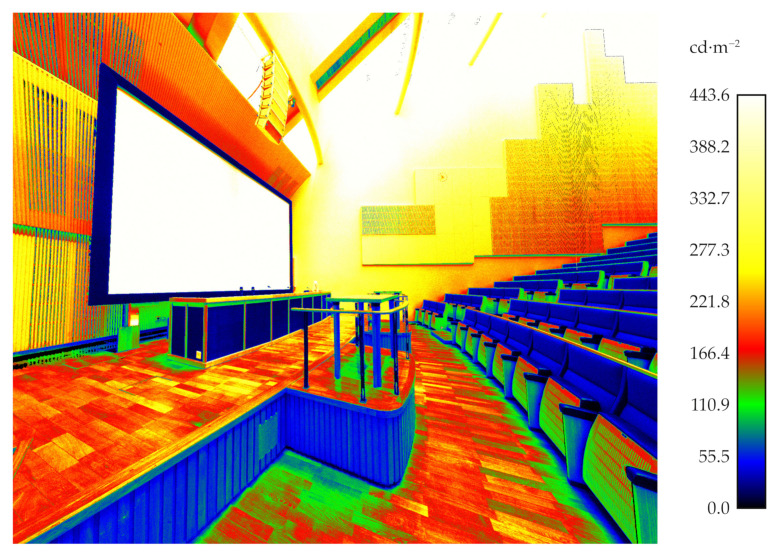
The luminance point cloud of scanning station 2.

**Figure 12:**
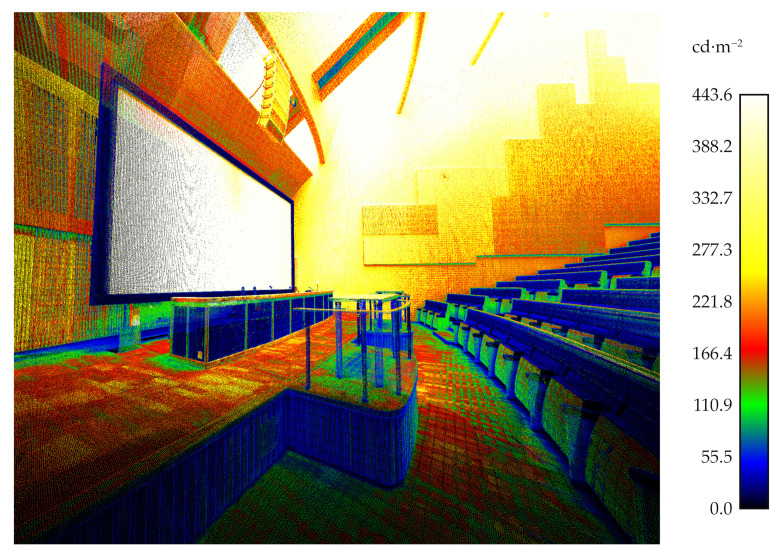
The subsampled luminance point cloud of all scanning stations.

The chosen test site was measured with a luminance-calibrated TLS. Figure 11 shows the luminance measurement obtained a single scan station projected onto 3D points, while Figure 12 illustrates seven merged luminance measurements subsampled to the octree level 12 as described in Section 2.3. The range of measured luminances was 0–443.6 cd·m${}^{-2}$. In the measured space, the measurement range covers most of the measurable surfaces. However, the luminance of the light sources and the surfaces around them were too high to be measured with the TLS used in this study.

#### 3.3.2. Sample Area Analysis of 3D Luminance Measurements

**Figure 13:**
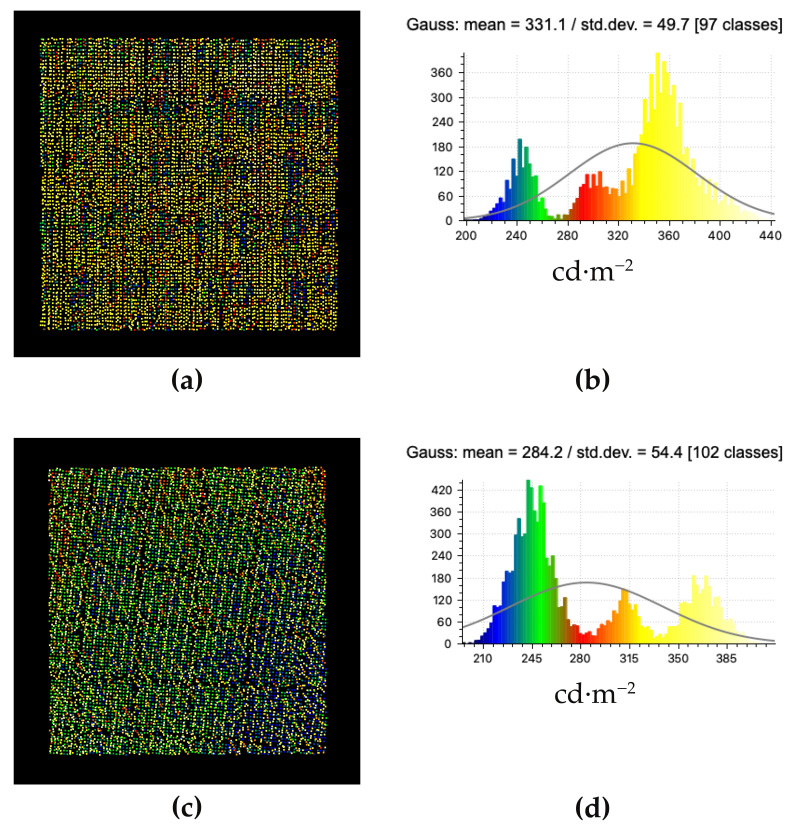
The point cloud (**a**) and its corresponding histogram (**b**) for the vertical sample area, and the point cloud (**c**) and its corresponding histogram (**d**) for the horizontal sample area.

Figure 13 illustrates the point clouds and their corresponding merged histograms for the vertical and horizontal sample areas (see Figure 8). Illustrations of each laser scan and their merged point clouds and corresponding histograms for both sample areas can be found in Appendix A.2.

Table 9 and Table 10 present the measured features and statistics for the vertical sample area and the horizontal sample area, respectively. The values presented are the median, Gaussian mean, minimum and maximum luminances, standard deviation, relative standard deviation, number of points, and angle between the surface normal and the measurement direction.

The sample areas show that, especially near the scanner, some scans are over-represented (Table 9 and Table 10). The number of points depends on the scanning angle and distance, so these features must be taken into account in the visual observation.

Table 11 presents the measured features and statistics for the sample areas A–G. The sample areas are from the merged luminance measurements subsampled to the octree level 12 as described in Section 2.3. The values presented are the median, Gaussian mean, minimum and maximum luminances, standard deviation, relative standard deviation, number of points, and angle between the surface normal and the measurement direction.

## 4. Discussion and Conclusions

### 4.1. Laboratory Measurements

We characterized the color and luminance measurement quality of a terrestrial laser scanner and we presented a workflow where an HDR image captured by a TLS instrument was converted into absolute luminance values. Compared to the reference, the TLS captured luminance values with an average absolute difference of 2.0 cd·m${}^{-2}$ and an average relative difference of 2.9% for the grayscale patches (No. 1–6). For all patches, the average absolute difference and average relative differences were 5.7 cd·m${}^{-2}$ and 7.5%, respectively. The relative difference between the TLS measurement and the reference measurement was notable for certain patches such as blue (46.7%) and cyan (22.3%). This indicates that certain heavily weighted spectra translate suboptimally into luminance values when using standard sRGB conversion factors. However, as we can characterize the channel-wise values for each patch in the X-Rite ColorChecker, we would be able to obtain conversion factors that would be more optimal for the camera in the TLS than the sRGB conversion factors. Optimized factors could possibly decrease the difference between the luminance values measured with the TLS and the reference values for the weighted spectra.

### 4.2. Field Measurements

We explored the possibilities of simultaneous laser scanning and luminance imaging through a case study. Thus, the level of automation increased in comparison with the previous luminance data and TLS point cloud integration, and the luminance data integrity and usability improved.

The dynamic range needed for luminance measurement depends on the application. The widest dynamic range is required when measuring nighttime outdoor environments, for example, road lighting. In order to measure the lowest end of mesopic luminances on the road surface to the glaring light source, a measurement range of 0.01 cd·m${}^{-2}$ to approximately 100,000 cd·m${}^{-2}$ would be needed. This is a little more than 23 f-stops. The system used in this study had an effective dynamic range of 4.3–443.6 cd·m${}^{-2}$ or a bit less than 9 f-stops. This dynamic range is almost sufficient to measure the luminance distribution of the surfaces in an indoor space but nowhere near wide enough for road lighting measurements. Moreover, it is a technologically difficult task to extend the dynamic range towards the low luminance levels. The sensors would have to be more sensitive yet have a better signal-to-noise ratio. Another solution is to apply HDR imaging with longer exposure times, which obviously makes measuring slower or less convenient.

For indoor applications, however, HDR imaging could be applied by adding images captured with shorter exposure times. This way, the dynamic range of a TLS could be extended to be sufficient for indoor measurement from the low-end surface luminances ( 1 cd·m${}^{-2}$) to the glaring light sources ( 100,000 cd·m${}^{-2}$). This upward extension of the dynamic range would enable the measurements needed when calculating the unified glare rating (UGR). Furthermore, it would be possible to measure the luminance of the light sources if the dynamic range of HDR imaging is wide enough.

To determine the location of the measuring device, terrestrial laser scanning allows the measurement angle to be defined for each measured point. The point can be assigned a location, color value, absolute luminance value, intensity, point normal, and angle between point normal and surface normal. This information can be used in the future to determine the properties of the scanned object, such as reflectivity and gloss. The angle between the scan station and the measured surface normal was not verified by any other method in this study. We considered the collected point cloud data accurate enough for angle measurement.

### 4.3. Limitations of TLS as a Luminance Photometer

Usually, a TLS instrument captures a panoramic image as a composite of several adjacent images that are overlapped and blended together. The technique is often called image stitching. The quality of the stitching is difficult to quantify, and we did not assess the inaccuracies of image stitching. However, the TLS instrument (Leica RTC360) could be more suitable if the uncertainty of the panoramic image stitching process was known and there was a possibility to maintain the bit depth of the measurement in the RGB-registered point clouds. As for now, registering the raw imaging bit depth into the point cloud requires manual effort.

Different TLS instruments employ various imaging sensor installations, such as completely separate camera systems operated from atop of the TLS instrument (e.g., Riegl [37]), integrated imaging sensors utilizing the same rotating mirror as the laser ranging sensor, or sets of cameras mounted in the instrument’s chassis, as in the applied Leica RTC360 scanner [3]. The realization of the imaging system affects the quality of produced panoramic images, e.g., through differences in parallax.

Contemporary TLS instruments are capable of obtaining rather high point densities and measurement speeds. For example, for the instrument applied in our work, the manufacturer reports a measuring speed of 2 million points per second and a point spacing of 3 mm at 10 m [31]. As a result, a single point cloud obtained with this instrument may contain up to approx. 200 million points [38]. A mapping campaign in a complex indoor environment may therefore well exceed a billion points. These data amounts present a technical challenge and require suitable storage systems to be applied in processing and distribution. Understandably, point cloud storage [39], distribution [40], and application [41] have become topical development tasks.

For assessing the color measurement of the TLS instrument, the 24 patch X-Rite ColorChecker Classic was applied. In order to improve the color measurement assessment, a color chart with 99 patches could be used as defined in ANSI/IES Method for Evaluating Light Source Color Rendition TM-30-20 [42].

### 4.4. Future Research Directions

In future studies, a method for determining the reflectivity of a surface can be developed as the locations of the measurements and the locations and luminances of the light sources are known. However, this method does not completely solve the reflectivity measurement. For more reliable reflectivity measurement, the light distribution of the light sources and the integration of light within the measurement space also need to be determined.

Simultaneous geometry and luminance measuring executed with a TLS can be applied in lighting design and lighting retrofitting. A 3D mesh model can be created from the measured point cloud. The mesh model can be converted into a CAD 3D model, which can be imported into lighting design software such as DIALux or Relux.

Since the scanner alone is not yet comparable in terms of image quality, the best result is obtained by combining terrestrial laser scanning and photogrammetry. As of yet, a TLS cannot replace conventional imaging luminance photometry in terms of luminance measurement. However, the TLS-based luminance measurement does not fall far behind. When the measurable luminance range is widened, the TLS luminance measurement would perform at a similar level as conventional imaging luminance photometry for indoor measurements and outdoor daytime measurements. Furthermore, both of these required improvements have been solved as individual technologies, but the advancements have not yet been implemented in a TLS. Hence, we are only a few steps away from luminance measurements being obtained as a side product of geometry measurement or vice versa. In TLS luminance measurement, the luminance data is registered into the measured geometry. This is a feature that is completely unobtainable using only conventional imaging luminance photometry.

As TLS point clouds capture the surrounding environment from all directions, their study requires different user interfaces than those used for navigating 2D image data sets. 3D point clouds can of course be studied on conventional displays, either with freely navigable 3D environments or—akin to panoramic images—by fixing the viewpoint and jumping from one measuring position to another. In complex indoor environments, immersive display devices, such as virtual reality head-mounted displays, offer a potentially more intuitive alternative for navigating complex virtual 3D environments. By leveraging game-engine technology, laser scanning point clouds can be brought into VR [43]. Adapting the point cloud visualization to the study of luminance data represents an obvious task for future development. 

## Figures and Tables

**Table 1:** Measurements and results performed in the study and their sections.

Laboratory measurements	Method:	Reference color target measurements: (Section 2.2 Luminance calibration of a terrestrial laser scanner; Section 2.3 Luminance data processing)
	Results:	Luminance calibration factor for TLS: (Section 3.1 Reference color target measurements; Section 3.2 Luminance measurement comparison and Appendix A.1 Color target)
Field measurements	Method:	TLS data of the study area: (Section 2.4 Case study) and the luminance calibration factor from laboratory measurements
	Results:	Absolute luminance point clouds: (Section 3.3 Case study and Appendix A.2 Sample areas)

**Table 2:** The colorimetric reference data for the ColorChecker Classic chart provided by X-Rite. X-Rite No. is the patch name used by X-Rite; L is the luminance value; a and b are color coordinates.

Patch No.	X-Rite No.	L	a	b
1	A4	95.19	−1.03	2.93
2	B4	81.29	−0.57	0.44
3	C4	66.89	−0.75	−0.06
4	D4	50.76	−0.13	0.14
5	E4	35.63	−0.46	−0.48
6	F4	20.64	0.07	−0.46
7	A3	28.37	15.42	−49.8
8	B3	54.38	−39.72	32.27
9	C3	42.43	51.05	28.62
10	D3	81.8	2.67	80.41
11	E3	50.63	51.28	−14.12
12	F3	49.57	−29.71	−28.32
13	A2	62.73	35.83	56.5
14	B2	39.43	10.75	−45.17
15	C2	50.57	48.64	16.67
16	D2	30.1	22.54	−20.87
17	E2	71.77	−24.13	58.19
18	F2	71.51	18.24	67.37
19	A1	37.54	14.37	14.92
20	B1	64.66	19.27	17.5
21	C1	49.32	−3.82	−22.54
22	D1	43.46	−12.74	22.72
23	E1	54.94	9.61	−24.79
24	F1	70.48	−32.26	−0.37

**Table 3:** The sample areas.

Sample Area	Material
A	wooden door
B	painted wood
C	painted concrete wall
D	painted vertical slatted timber
E	painted vertical slatted timber
F	painted horizontal slatted timber
G	painted concrete wall
Vertical	textile-covered acoustic board
Horizontal	wooden table

**Table 4:** The sRGB values (16-bit) of the X-Rite ColorChecker Classic board, calculated from the spectra measured with the spectroradiometer, and scaled to match the X-Rite nominal values.

Patch No.	R	G	B
1	62,782	62,269	56,751
2	41,236	41,525	38,895
3	25,391	25,701	23,993
4	13,472	13,375	12,370
5	6498	6633	6285
6	2699	2606	2414
7	2660	3735	20,103
8	4945	20,983	4284
9	29,753	3094	2760
10	57,323	40,884	285
11	35,392	6377	20,273
12	−447	16,836	24,888
13	50,372	14,128	1888
14	4941	7354	26,861
15	38,649	6226	8016
16	7631	3249	9593
17	24,366	36,005	2981
18	53,556	26,190	1328
19	12,565	6284	4060
20	38,994	20,567	14,321
21	8588	13,919	22,313
22	7652	11,057	3514
23	16,275	14,978	28,129
24	9578	36,325	26,761

**Table 5:** The laser scanner luminance measurements compared to a spectroradiometer. The table shows the differences and relative differences between the reference luminance measured with a spectroradiometer and the luminance measured with a TLS. The luminance measured with a TLS was calculated by linear regression and by linear regression and noise removal.

Patch No.	A	B	C	Diff. (A,C)	Relative Diff. (A,C)	D	Diff. (A,D)	Relative Diff. (A,D)
1	329.8	48,753.6	333.2	3.4	1.0%	330.8	1.0	0.3%
2	219.6	32,784.2	224.1	4.4	2.0%	221.0	1.4	0.6%
3	135.8	20,786.2	142.1	6.3	4.7%	138.5	2.8	2.0%
4	70.9	11,449.6	78.3	7.4	10.4%	74.4	3.5	4.9%
5	35.0	6165.6	42.1	7.1	20.4%	38.1	3.0	8.7%
6	13.9	2665.2	18.2	4.3	31.1%	14.0	0.1	0.7%

A: Spectroradiometer value in cd·m${}^{-2}$. B: Laser scanner 16-bit value, average of five scans. C: Laser scanner luminance value in cd·m${}^{-2}$, obtained by linear regression. D: Laser scanner luminance value in cd·m${}^{-2}$, obtained by linear regression and noise removal.

**Table 6:** The consistency of images in five consecutive TLS scans.

Patch No.	#1	#2	#3	#4	#5	avg	STD	RSD
**1**	48,403	48,073	48,437	49,867	48,988	48,753.6	703.7	1.44%
**2**	32,658	32,384	32,803	33,317	32,759	32,784.2	339.5	1.04%
**3**	20,718	20,572	20,987	21,028	20,626	20,786.2	209.2	1.01%
**4**	11,396	11,394	11,512	11,601	11,345	11,449.6	104.5	0.91%
**5**	6144	6112	6180	6292	6100	6165.6	77.2	1.25%
**6**	2638	2636	2680	2712	2660	2665.2	31.7	1.19%

**Table 7:** The relative luminance values calculated from the TLS 16-bit linear images compared to different sets of reference values. The table presents the values ordered according to the reference color target (Figure 2).

TLS 16-Bit Linear Images Compared to the ReferenceValues Measured with a Spectroradiometer					
Relative luminance values calculated from the TLS 16-bit linear images					
8418.6	24,482.7	15,137.8	10,691.0	17,647.0	31,555.7
19,673.6	10,007.0	13,167.5	5486.0	30,092.9	26,973.7
7559.0	18,091.8	37,820.5	37,820.5	13,644.1	17,318.7
61,969.5	41,645.5	14,543.5	14,543.5	7838.0	3379.1
Linear spectroradiometer values calculated and scaled from the measured spectra					
7458.4	24,033.5	13,392.0	9788.6	16,203.3	29,948.3
20,950.0	8249.2	13,248.4	4638.9	31,146.1	30,213.0
4688.5	16,367.8	8737.3	41,447.7	13,549.	13,742.7
61,979.3	41,273.9	25,512.0	13,322.6	6578.9	2611.9
Relative difference between the values measured with a TLS and a spectroradiometer					
12.9%	1.9%	13.0%	9.2%	8.9%	5.4%
6.1%	21.3%	0.6%	18.3%	3.4%	10.7%
61.2%	10.5%	7.2%	8.8%	0.7%	26.0%
0.0%	0.9%	3.5%	9.2%	19.1%	29.4%
				Average:	12.0 %

**Table 8:** The adjusted TLS luminance measurements compared to the reference values measured with a spectroradiometer. The table presents the values ordered according to the reference color target (Figure 2).

TLS Luminance Measurements Compared to the Reference Values Measuredwith a Spectroradiometer					
Absolute adjusted luminance values measured with a TLS					
41.1	127.9	77.7	53.4	91.3	166.4
101.7	50.1	66.7	25.4	158.1	141.1
36.8	93.4	46.2	199.6	69.5	89.6
330.9	221.0	138.4	74.3	38.1	14.0
Absolute adjusted luminance values measured with a spectroradiometer					
39.7	127.8	71.4	52.0	86.4	159.4
111.3	44.1	70.5	24.7	165.5	160.5
25.1	87.0	46.4	220.2	72.2	73.3
329.8	219.6	135.8	70.9	35.0	13.9
Absolute difference					
1.5	0.1	6.4	1.4	5.0	7.1
9.6	6.0	3.7	0.7	7.4	19.4
11.7	6.4	0.3	20.6	2.7	16.3
1.1	1.3	2.7	3.4	3.1	0.1
				Average:	5.7
Relative difference					
3.7%	0.1%	8.9%	2.7%	5.8%	4.4%
8.6%	13.7%	5.3%	2.8%	4.5%	12.1%
46.7%	7.4%	0.6%	9.3%	3.7%	22.3%
0.3%	0.6%	2.0%	4.9%	8.8%	0.4%
				Average:	7.5 %

**Table 9:** The vertical sample area: median luminance, Gaussian mean luminance, minimum luminance, maximum luminance, standard deviation, relative standard deviation, number of points, and angle between the surface normal and the measurement direction.

No.	L_median_	L_mean_	L_min_	L_max_	L_STD_	L_RSD_	Points	Angle
1	243.0	242.2	198.1	279.7	11.0	4.5%	10,066	26${}^{\circ }$
2	346.6	345.7	278.3	402.0	13.8	4.0%	9460	17${}^{\circ }$
3	379.9	349.9	296.9	443.0	22.7	6.0%	10,184	66${}^{\circ }$
4	358.7	358.2	314.7	411.7	12.9	3.6%	8310	59${}^{\circ }$
5	334.7	334.3	283.8	388.9	15.6	4.7%	3537	69${}^{\circ }$
6	299.4	300.0	265.9	341.4	10.9	3.6%	6273	36${}^{\circ }$
7	353.8	353.4	300.9	407.9	13.9	3.9%	8805	18${}^{\circ }$
All	346.3	330.7	198.1	443.0	48.9	14.8%	56,635	17–69${}^{\circ }$
All_subsampled_	346.5	331.1	198.1	442.9	49.7	15.0%	9295	17–69${}^{\circ }$

**Table 10:** The horizontal sample area: median luminance, Gaussian mean luminance, minimum luminance, maximum luminance, standard deviation, relative standard deviation, number of points, and angle between the surface normal and the measurement direction. Scans 2 and 3 were left with no observations due to the large measurement angle.

No.	L_median_	L_mean_	L_min_	L_max_	L_STD_	L_RSD_	Points	Angle
1	244.9	244.4	194.8	346.3	14.4	5.9%	7374	63${}^{\circ }$
2	-	-	-	-	-	-	-	87${}^{\circ }$
3	-	-	-	-	-	-	-	88${}^{\circ }$
4	369.5	369.7	311.6	410.0	12.4	3.4%	3617	81${}^{\circ }$
5	310.2	310.0	266.0	356.7	13.0	4.2%	2264	76${}^{\circ }$
6	367.5	367.9	315.2	419.5	14.4	3.9%	2620	76${}^{\circ }$
7	428.1 *	427.9 *	399.1 *	443.5 *	5.1 *	1.2% *	6336	79${}^{\circ }$
All	302.6	302.7	194.8	419.5	59.2	19.5%	15,875	63–88${}^{\circ }$
All_subsampled_	257.3	284.2	194.8	419.5	54.4	19.1%	10,367	63–88${}^{\circ }$

* Scan number 7 was partly overexposed and therefore omitted from the merged clouds.

**Table 11:** The sample areas A–G: median luminance, Gaussian mean luminance, minimum luminance, maximum luminance, standard deviation, relative standard deviation, number of points, and angle between the surface normal and the measurement direction.

Sample Area	L_median_	L_mean_	L_min_	L_max_	L_STD_	L_RSD_	Points	Angle
A	90.7	89.3	44.0	128.8	14.0	15.7%	8468	16–69${}^{\circ }$
B	194.3	192.5	98.0	255.0	26.2	13.6%	13,247	11–51${}^{\circ }$
C	369.2	369.3	277.9	443.4	35.2	9.5%	9376	18–63${}^{\circ }$
D	159.0	165.5	42.3	336.4	60.9	36.8%	20,158	11–79${}^{\circ }$
E	268.3	268.1	117.0	430.9	56.3	21.0%	19,172	9–65${}^{\circ }$
F	178.6	174.4	105.0	233.2	24.3	14.0%	12,208	15–65${}^{\circ }$
G	231.2	223.9	150.6	276.9	29.3	13.1%	9836	14–63${}^{\circ }$
Vertical	346.5	331.1	198.1	442.9	49.7	15.0%	9295	17–69${}^{\circ }$
Horizontal	257.3	284.2	194.8	419.5	54.4	19.1%	10,367	63–88${}^{\circ }$

## Data Availability

The data presented in this study are openly available in Zenodo at 10.5281/zenodo.4743890, reference number [44].

## Appendix A. Measurement Details

### Appendix A.1. Color Target

Table A1 and Table A2 show the 16-bit values for each R, G, and B channel measured with the TLS and the spectroradiometer respectively. Values are linear and scaled to be comparable. Table A3 presents the relative differences between the measurements.

**Table A1:** Linear TLS measurement scaled to be comparable with the X-Rite color target values for each channel R, G, and B.

Linear TLS Measurements					
R					
10,692.7	30,952.0	13,659.6	10,150.2	18,788.2	23,719.4
30,721.9	9517.4	24,409.8	75,61.3	27,007.0	35,455.9
6262.7	13,528.1	18,500.5	43,229.6	23,587.9	12,114.5
62,099.9	41,784.1	26,448.1	14,719.9	7959.9	3515.6
G					
7979.8	23,218.5	14,811.0	11,299.7	16,310.0	33,972.4
17,776.4	8586.8	10,183.6	4419.7	33,092.5	26,607.6
6721.2	20,359.0	7095.9	39,333.0	10,118.4	17,906.8
62,077.7	41,613.9	26,379.5	14,525.8	7776.2	3321.9
B					
6069.3	17,956.2	22,728.0	6253.3	27,530.8	30,691.4
5933.8	25,516.7	9621.1	9938.7	9466.1	5623.9
19,675.9	9071.1	4934.5	6909.8	19,288.6	26,818.1
60,513.9	41,550.7	26,353.4	14,199.2	8091.1	3544.0

**Table A2:** Linear spectroradiometer measurement scaled to be comparable with the X-Rite color target values for each channel R, G, and B.

Linear Spectroradiometer Measurements					
R					
12,564.9	38,994.4	8588.3	7652.1	16,275.1	9578.0
50,372.5	4940.8	38,649.1	7630.9	24,366.3	53,555.9
2660.2	4944.7	29,753.2	57,323.2	35,391.7	−446.7
62,781.8	41,236.4	25,391.0	13,471.6	6497.8	2699.1
G					
6283.6	20,566.6	13,919.4	11,057.2	14,978.0	36,325.3
14,128.2	7353.7	6226.0	3249.3	36,004.7	26,190.2
3735.4	20,983.3	3093.5	40,883.9	6377.4	16,835.5
62,268.6	41,525.3	25,701.3	13,374.6	6632.7	2605.9
B					
4059.6	14,321.4	22,313.1	3513.8	28,129.3	26,760.9
1888.1	26,861.4	80,16.2	9593.0	2980.7	1327.7
20,103.2	4284.2	2760.3	285.3	20,272.9	24,888.5
56,751.4	38,894.6	23,993.5	12,369.5	6284.7	2413.8

**Table A3:** The absolute values of relative differences between the linear TLS and spectroradiometer measurements for each channel R, G, and B.

The Relative Differences					
R					
14.9%	20.6%	59.0%	32.6%	15.4%	147.6%
39.0%	92.6%	36.8%	0.9%	10.8%	33.8%
135.4%	173.6%	37.8%	24.6%	33.4%	2811.9%
1.1%	1.3%	4.2%	9.3%	22.5%	30.2%
				average:	76.4%
G					
27.0%	12.9%	6.4%	2.2%	8.9%	6.5%
25.8%	16.8%	63.6%	36.0%	8.1%	1.6%
79.9%	3.0%	129.4%	3.8%	58.7%	6.4%
0.3%	0.2%	2.6%	8.6%	17.2%	27.5%
				average:	23.1%
B					
49.5%	25.4%	1.9%	78.0%	2.1%	14.7%
214.3%	5.0%	20.0%	3.6%	217.6%	323.6%
2.1%	111.7%	78.8%	2322.1%	4.9%	7.8%
6.6%	6.8%	9.8%	14.8%	28.7%	46.8%
				average:	149.9%

### Appendix A.2. Sample Areas

Table A4 and Table A5 show the luminance values of each sample area for each scan as well as illustrations of each laser scan and its merged point clouds and corresponding histogram for both of the samples. The deviations observed between the different scans were caused by different viewing angles and possible changes in luminance between the different scans.

**Table A4:** The vertical sample area: included scans (single scan stations “1–7”, merged scans “All”, and the merged scan subsampled with octree level 12), sample area visualization, histogram of luminances, number of points, and angle between the surface normal and the measurement direction.

No.	Sample Area	Histogram	Points	Angle
1			10,066	26${}^{\circ }$
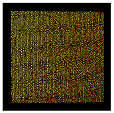
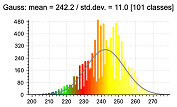
2			9460	17${}^{\circ }$
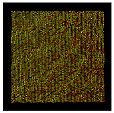
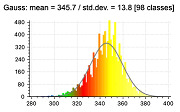
3			10,184	66${}^{\circ }$
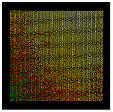
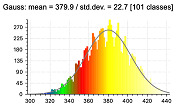
4			8310	59${}^{\circ }$
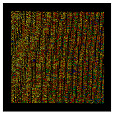
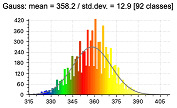
5			3537	69${}^{\circ }$
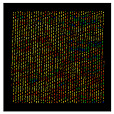
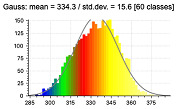
6			6273	36${}^{\circ }$
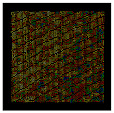
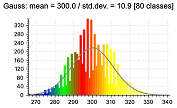
7			8805	18${}^{\circ }$
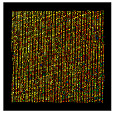
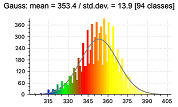
All			56,635	17–69${}^{\circ }$
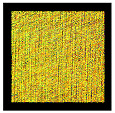
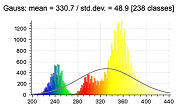
All_subsampled_			9295	17–69${}^{\circ }$
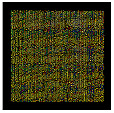
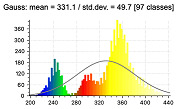

**Table A5:** The horizontal sample area: included scans (single scan stations “1–7”, merged scans “All”, and merged scan subsampled with octree level 12), sample area visualization, histogram of luminances, the number of points, and the angle between the surface normal and the measurement direction. Scans 2 and 3 were left with no observations due to the large measurement angle. Scan number 7 was partly overexposed and therefore omitted from the merged clouds.

No.	Sample area	Histogram	Points	Angle
1			7374	63${}^{\circ }$
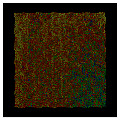
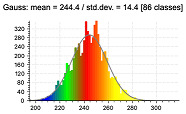
2	-	-	-	87${}^{\circ }$
3	-	-	-	88${}^{\circ }$
4			3617	81${}^{\circ }$
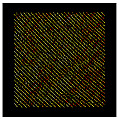
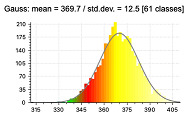
5			2264	76${}^{\circ }$
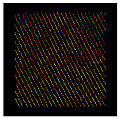
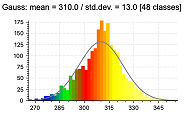
6			2620	76${}^{\circ }$
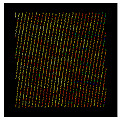
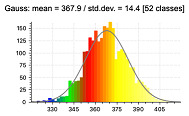
7			6336	79${}^{\circ }$
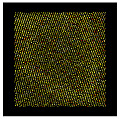
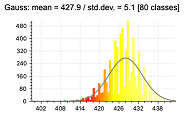
All			15,875	63–88${}^{\circ }$
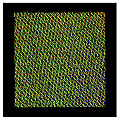
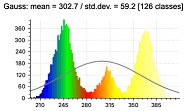
All_subsampled_			10,367	63–88${}^{\circ }$
